# Neutrophils as a Therapeutic Target in Cancer

**DOI:** 10.3389/fimmu.2019.01710

**Published:** 2019-07-19

**Authors:** Zvi Granot

**Affiliations:** Department of Developmental Biology and Cancer Research, The Institute for Medical Research Israel-Canada, The Hebrew University-Hadassah Medical School, Jerusalem, Israel

**Keywords:** neutrophils, cancer, metastasis, tumor microenvironment, therapy

## Abstract

Neutrophils are the most abundant population of white blood cells in the human circulation. They are terminally differentiated myeloid cells which were traditionally associated with fighting infections and inflammatory processes. While this perception of neutrophils is still widely prevalent, in the past decade it has become clear that neutrophils also play a critical role in tumor growth and progression. The unique tumor microenvironment, consisting of the non-malignant stroma that surrounds tumor cells, is shaped by numerous cues emanating from both tumor cells and stromal cells which support the growing tumor. Various immune cells, including neutrophils, make up a significant proportion of the tumor stroma. Immune cells exist for the protection of the host against various threats including the detection and elimination of cancerous cells. However, in the context of cancer immune cells are often coerced into a tumor supportive phenotype. This is also the case for neutrophils, which are often described to possess tumor promoting properties and to associate with poorer prognosis. The fact that neutrophils may contribute to tumor growth and progression suggests they may be targets for anti-cancer therapies. This review discusses the various functions neutrophils may play in cancer and the possibility of targeting these functions as a novel mode of immunotherapy.

## Distinct Neutrophil Subsets or a Functional Spectrum?

Neutrophils are phagocytes which play a key role in protection of the host against microbial infections as well as taking a critical part in inflammatory processes. In the context of cancer, neutrophils were also shown to play other, non-conventional roles, and may either promote (1, 2) or limit tumor growth (3–5). The conflicting reports regarding neutrophil function in cancer suggest that like other cells of the immune system, neutrophils may be divided into distinct subsets. However, until recently neutrophils were viewed as a homogeneous population of terminally differentiated cells. Still, in a recent study we were able to show that neutrophils in the context of cancer may be divided into 3 subsets—Normal Density Neutrophils (NDN), mature and immature Low Density Neutrophils (LDN) (6). We were able to associate cytotoxic anti-tumor properties with NDN and immunosuppressive pro-tumor properties with LDN (6). In fact, neutrophils subsets distinguishable by their density were found in a wide range of clinical scenarios and are not only associated with cancer (7). Unlike cells of the adaptive immune system, which can be easily defined based on surface expression of unique markers, such surface markers are not well-characterized for neutrophils. In fact, several studies suggested possible markers but these still need to be better validated (8, 9). Still, neutrophil subsets may be distinguished according to their different physical properties (6) and there is increasing evidence for the existence of various neutrophil subsets which may be defined by their functionality. The lack of validated surface markers, together with their short half-life, makes neutrophils very difficult to study. Further, although specific functionally distinct subsets may be identified, it is still not clear whether these are truly specific subsets or are they simply found on extreme ends of a functional spectrum. That said, the accumulating data regarding neutrophil function in cancer highlights various functional aspects that may be targeted or modified to benefit patients. Following is an account of neutrophil functions and characteristics in the context of cancer and a discussion of how and whether targeting these aspects is feasible or beneficial for cancer therapy.

## Neutrophil to Lymphocyte Ratio

Neutrophils are notorious for their tumor promoting properties (1, 2, 10). First and foremost, high neutrophil numbers, otherwise manifested as the Neutrophil to Lymphocyte Ratio (NLR), represent a poor prognostic factor. This was found to be applicable to breast, colon, liver, and many other types of cancer (10). The reasons for the increase in neutrophil numbers are not always fully understood. Some tumors express high levels of colony stimulating factors (i.e., G-CSF and GM-CSF) which may account for the increase in mobilized neutrophils. Other tumors are in a state of smoldering inflammation which may also drive the increase in neutrophil numbers.

NLR relates to the numbers of circulating neutrophils, however, the extent of neutrophil infiltration of tumors also appears to have an adverse prognostic value (11). High neutrophil infiltration is associated with poor prognosis, advanced stage cancer and lower recurrence free survival (12–16). Some evidence suggest that high NLR may correlate with the number of tumor associated neutrophils (17). However, this needs to be further evaluated.

These observations raise a question regarding the possible targeting of neutrophils as a means for better patient outcome. Neutrophils are critical for anti-microbial protection, the option of eliminating neutrophils as a therapeutic strategy cannot be seriously considered since neutropenia is a life threatening condition. A possible alternative would be the depletion of specific neutrophil subpopulations while sparing those subpopulations essential for anti-microbial protection (see above). Unfortunately, although the existence of distinct neutrophil subsets in cancer has been convincingly demonstrated, our understanding of neutrophil subsets and the features making them distinct is still lacking. Specifically, as long as there are no clear markers to distinguish specific subsets, eliminating specific subsets for therapeutic purposes is impossible.

## Pro-tumor Neutrophil Functions

### Angiogenesis

**Figure 1:**
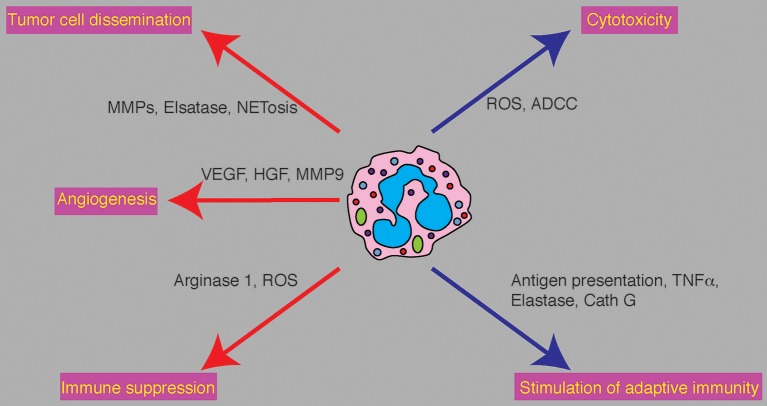
Neutrophil functions in cancer and potential therapeutic targets. Neutrophils play various and conflicting roles in cancer. Tumor promoting functions (red arrows) and anti tumor functions (blue arrows) are executed by specific molecular mediators. Tumor promoting propeties: Neutrophils promote tumor cell dissemination by degradation of the ECM at the primary and premetastatic sites and promote tumor cell seeding by deploying NETs. Promotion of angiogenesis is mediated by secretion of VEGF and HGF and the release of angiogenic factors from the ECM by neutrophil derived MMP9. Neutrophil mediate immune supprssion via the secretion of ROS and Arginase 1 to limit T cell dependent anti-tumor immunty. Anti-tumor properties: Neutrophils limit tumor growth and metastatic progression by eliminating tumor cells either directly or via antibody dependent mechanisms. Neutrophils can stimulate anti-tumor adaptive immune by acting as antigen presenting cells, secretion of TNFα, secretion of Elastase and secretion of Cathepsin G (Cath G).

The angiogenic switch that characterizes a transition toward a more aggressive tumor phenotype is regulated by the expression of angiogenic factors such as VEGF (18). As such, targeting angiogenesis should serve to limit tumor growth. This indeed turned out to be the case to a limited extent and in certain types of cancer (19). When looking for the source of angiogenic factors in the tumor microenvironment, neutrophils, together with other stromal cells, were shown to provide proangiogenic factors and actively promote tumor angiogenesis. Specifically, neutrophils were shown to provide MMP9, VEGF and HGF (Figure 1). Furthermore, neutrophils were shown to provide factors that circumvent common anti-angiogenic therapies targeting VEGF (20). Taken together, these observations highlight a key role for neutrophils in propagating tumor angiogenesis and suggest that targeting of neutrophil mediated angiogenesis, or targeting of the angiogenic neutrophil subpopulation (if such subpopulation indeed exists), may be used as an anti-angiogenic therapeutic approach.

### Tumor Cell Dissemination

Metastasis is the final and lethal stage in cancer progression. For tumor cells to metastasize they need to acquire unique features that support the transition from the primary site, their survival in the circulation and the successful metastatic seeding in a distant organ. In this context neutrophils were shown to play various roles to promote the intravasation of tumor cells (MMPs and neutrophil elastase, Figure 1), their survival in the circulation (21), their adherence to the endothelium at the future site of metastasis (priming of the premetastatic niche and NETs) and the process of extravasation (Figure 1). Recently neutrophils were also shown to play a critical role in the awakening of dormant tumor cells and the initiation of metastases growth (22). Targeting of neutrophil function in each of these stages of metastatic dissemination may have significant implications on metastatic progression. This is elegantly demonstrated in a recent study by Albrengues et al. (22) who show that NETs are required for promoting the exit from dormancy and the establishment of marcometastases. This finding is noteworthy since it proposes a clinical scenario where intervention is still possible, i.e., administration of DNAse to eliminate NETs to maintain tumor cells dormant and prolong distant metastasis free survival.

### Immune Suppression

The term Myeloid Derived Suppressor Cells (MDSC) encompasses a wide range of myeloid cells which possess immunosuppressive properties. In the context of cancer, these cells have the capacity to suppress cytotoxic T cells and promote immune evasion. The broadness of the MDSC umbrella also covers neutrophils but since it is a relatively well-defined population the term suppressive neutrophils is more accurate. We have previously shown that immunosuppressive neutrophils are propagated to promote the resolution of an inflammatory process. It seems that a similar rationale is employed in the context of cancer—the propagation of immunosuppressive neutrophils serves the resolution of tumor associated inflammation. However, since the tumor is in a continuous state of inflammation that does not resolve, suppressive neutrophils are mobilized excessively to the point where they become the dominant subpopulation of neutrophils. Under these conditions the overall neutrophil contribution is pro-tumorigenic. Immunosuppressive neutrophils (often referred to as G-MDSC) contain large amounts of arginase I (Figure 1) which suppresses T cell proliferation through deprivation of L-arginine (23, 24). Immunosuppressive neutrophils were also shown to generate high levels of hydrogen peroxide (Figure 1) and thus block T cell proliferation (25, 26). These observations provide insight into the role played by neutrophils that are maintaining an immunosuppressive tumor microenvironment and highlight their role in facilitating metastatic spread through suppression of adaptive immune components (6, 25, 27). These observations suggest that administration of immunotherapies concomitant with blocking of neutrophil-mediated immunosuppression may further potentiate anti-tumor adaptive immunity. This notion was in fact demonstrated in two separate studies; the first showing that blocking of c-MET in neutrophils improves the efficacy of immunotherapy by limiting the recruitment of immunosuppressive neutrophils (28). The second study, recently published by Veglia et al. (29) shows that FATP2 deficient neutrophils lose their immunosuppressive properties leading to a significant delay in tumor progression.

## Anti Tumor Neutrophil Functions

### Neutrophil Cytotoxicity

While most of the data regarding neutrophil function in cancer supports a pro-tumorigenic role, neutrophil may also eliminate cancerous cells and limit metastatic seeding. Unlike other neutrophil properties discussed above, neutrophil cytotoxicity requires a high level of specificity. Neutrophils need to be activated, they need to be attracted to tumor cells, they must identify tumor cells as a target, they must form physical contact with tumor cells and must secrete cytotoxic mediators (H_2_O_2_) to induce tumor cell apoptosis (Figure 1). Neutrophil recognition of tumor cells may be mediated either directly [RAGE-Cathepsin G (30)] or in an antibody dependent fashion [ADCC (31)]. In addition, tumor cells must be susceptible to neutrophil cytotoxicity (i.e., express the H_2_O_2_-dependent TRPM2 Ca^2+^ channel) for neutrophils to exert this favorable function (32). It seems that although cytotoxic neutrophils may be detected throughout the course of the disease, neutrophil cytotoxicity is mostly evident in early stages of tumor progression. This is most likely due to suppressive conditions that govern the tumor microenvironment. Since TRPM2 expression in tumor cells varies, not all tumor cells are equally susceptible to neutrophil cytotoxicity. Neutrophil resistant tumor cells should be targeted by other means. However, preventing the transition form HDN to LDN (perhaps by blocking TGFβ activity) should enhance the proportion of anti-tumor neutrophils and may be considered as a possible anti-cancer therapy. Further, the transfusion of cytotoxic neutrophils, although somewhat challenging, is actively being evaluated (*Lift BioSciences*).

### Stimulation of Adaptive Immune Responses

The notion that adaptive immunity is the major effector in anti-tumor immune responses is well-accepted. However, there is evidence supporting a role for neutrophils in this respect too. For example, neutrophils were shown to interact with T cells and are required for proper anti-tumor CD4^+^ and CD8^+^ T-cell responses (33–36). In fact, neutrophils were shown to present antigens and provide accessory signals for T cell activation (37, 38). In addition, N1 tumor associated neutrophils were shown to require T-cells for their anti-tumor activity at the primary site, which may indicate possible stimulation of T cells by neutrophils (33). Finally, neutrophils are able to recruit and activate T-cells via secretion of cytokines, including TNFα, Cathepsin G and neutrophil elastase (27) (Figure 1).

## Context Dependent Neutrophil Function

Neutrophils may present with either tumor promoting or tumor limiting properties. It is not yet clear whether this is a manifestation of distinct subsets or the extreme ends of a wide functional spectrum. Regardless, neutrophils are the first responders of the immune system and as such are equipped with a wide variety of receptors. This makes neutrophils highly responsive to cues in their microenvironment and may explain why neutrophils function one way at the primary tumor and in a completely different way in the pre-metastatic niche. Indeed, neutrophil function was found to be dramatically modified by factors such as TGFβ and type I interferons.

### TGFβ

TGFβ is a highly versatile molecule which may act as both tumor suppressor and oncogene. However, when examining the effect it exerts on neutrophil function in cancer it is regarded as pro tumoral. Fridlender et al. (33) were the first to show that TGFβ in the tumor microenvironment acts to block neutrophil cytotoxicity. In this study they also coined the “N1” anti-tumor and “N2” pro-tumor terminology to describe neutrophil function in cancer. Their study showed that TGFβ both blocked the anti-tumor function of neutrophils and restricted their entry into the tumor (33). Later studies provided better insight into the effect of TGFβ on neutrophil function in cancer. First, TGFβ directly blocks the production of H_2_O_2_, a key mediator of neutrophil cytotoxicity, by activated neutrophils. Second, TGFβ was found to block the migration of tumor neutrophils toward tumor cells. And third, TGFβ was found to change the ratio between HDN and LDN (see above). Together, these observations demonstrate that TGFβ not only blocks the favorable anti-tumor functions of neutrophils, it also increases the proportion of tumor promoting neutrophils thereby supporting tumor growth. Since TGFβ is abundant at the primary and metastatic tumors, neutrophil cytotoxicity is not evident in these sites but rather the pro-tumor functions are manifested. In contrast, during the early stages of metastatic dissemination, circulating tumor cells arriving to the future site of metastasis are not protected by high levels of TGFβ and are susceptible to neutrophil cytotoxicity. Hence neutrophil cytotoxicity is evident at the time of metastatic seeding and possibly at early stages of tumorigenesis but not in the microenvironment of an established tumor.

### IFNs

Type I interferons have an effect on neutrophil function that opposes that of TGFβ. IFNs were first identified as having anti-viral functions and later on were also found to play an anti-tumorigenic role. IFNs mediate an anti-tumor immune response by activating various immune cells (39). On top of modulating the function of lymphocytes and macrophages, IFN-β was found to suppress the expression of proangiogenic factors, such as VEGF and MMP9, thereby limiting tumor growth (40). In addition to modifying the expression of protumorigenic factors, IFN-β enhances the recruitment of neutrophils and their life span in primary tumors (41, 42). Finally, type I IFN activity was found to inhibit neutrophil-mediated priming of a receptive premetastatic niche (43).

Together, these observations support the notion that neutrophil function in cancer is heavily dictated by the specific microenvironment. More importantly, these data suggest that rather than modifying the function of neutrophils or depleting specific subsets, one may achieve a therapeutic benefit mediated by neutrophils via modulation of the tumor microenvironment. Essentially, blocking TGFβ activity or enhancing IFNs activity at the tumor microenvironment should facilitate neutrophil anti-tumor cytotoxicity and may be considered as a mode of anti-tumor immunotherapy.

## Concluding Remarks

Neutrophils are essential for host protection against microbial infections and as such cannot be eliminated as a mode of therapy. However, the progress made in recent years highlighting the fact that neutrophils are not a homogeneous population of cells, opens new opportunities for targeting neutrophils as a mode of cancer therapy. Better characterization of neutrophils, their different subsets and distinct functions may serve to specifically deplete harmful populations and enhance neutrophils' favorable functions. However, taking into account the fast rate of neutrophil replenishment, this strategy will require continuous administration of antibodies. This therapeutic approach is not without risk and previous studies using antibodies to deplete neutrophils show that ultimately the depleting antibodies lose their efficacy.

A different strategy for the manipulation of neutrophil function in cancer is via the modulation of the tumor microenvironment in a fashion that would permit neutrophil anti-tumor functions. Indeed, using small molecules to block TGFβ showed a dramatic effect on tumor growth that was dependent on neutrophils. Furthermore, Novitskiet al. demonstrated that tumor growth and metastatic spread are blocked when using a mouse model of myeloid-specific deletion of TGFβR2 (44). Together, these observations suggest that modifying TGFβ activity in neutrophils *in vivo* may be sufficient for stimulating a robust anti-tumor response. That said, current therapies targeting TGFβ signaling prove to be toxic and are not tolerated well. A possible alternative for circumventing the toxicity of systemic administration of small molecule TGFβ blockers is a more targeted approach. Future therapies using neutrophil specific drug delivery may serve to harness neutrophils toward fighting cancer. Such technology is yet to be developed.

## Author Contributions

The author confirms being the sole contributor of this work and has approved it for publication.

### Conflict of Interest Statement

The author declares that the research was conducted in the absence of any commercial or financial relationships that could be construed as a potential conflict of interest.
